# MiRNA Profiling in Plasma Exosomes of Pregnant Cows on Day 16

**DOI:** 10.3390/biology15110863

**Published:** 2026-05-30

**Authors:** Shijie Lyu, Yaying Zhai, Manru Luan, Fuying Chen, Xiaoting Zhu, Yajie Feng, Zhihui Qiao, Qiaoting Shi, Eryao Wang

**Affiliations:** 1 Institute of Animal Husbandry Science, Henan Academy of Agricultural Sciences, Zhengzhou 450002, Chinazhaiyaying@outlook.com (Y.Z.); manruluan0108@163.com (M.L.); fychen2004@sina.com (F.C.); fyjivy@163.com (Y.F.); 13783424679@139.com (Z.Q.); sqtsw@126.com (Q.S.); 2 The Shennong Laboratory, Zhengzhou 450000, China; 3 College of Animal Science, Henan Agricultural University, Zhengzhou 450046, China

**Keywords:** bovine, plasma exosome, miRNA profiles, different expression, pregnancy

## Abstract

Plasma exosomes have been identified to be involved in embryo development and implantation. In this study, the plasma exosomes of seven pregnant cows on day 16 and seven non-pregnant cows were collected. Exosome miRNA profiles were revealed by RNA sequencing technology and the abundance of exosome miRNA between them were compared. Seven miRNAs were significantly differently expressed (*p*-value < 0.05, |log2(FoldChange)| > 1), with five miRNAs upregulated and two downregulated in the pregnant cow group. The most significant miRNA was bta-miR-136 (downregulated in pregnant cows, *p*-value < 0.005). A total of 5828 target genes were computationally predicted for the seven miRNAs. GO and KEGG enrichment analyses showed these targets were significantly involved in several biological processes and pathways associated with embryo implantation and endometrial development, such as cell adhesion, cell junction, focal adhesion, Ras signaling pathway and Rap1 signaling pathway. This study identifies seven differentially expressed plasma exosomes miRNAs on day 16 of pregnancy in cows, with bta-miR-136, bta-miR-199b, and bta-miR-382-3p emerging as candidate miRNAs for future studies on maternal–fetal communication and embryo implantation.

## Simple Summary

We analyzed plasma exosomal miRNAs in cows on day 16 of gestation. Comparison of seven pregnant and seven non-pregnant animals revealed seven differentially expressed miRNAs, of which five were upregulated and two downregulated in the pregnant group. Among these, bta-miR-136 showed the greatest increase. Functional analyses suggest that the target genes of these miRNAs are enriched in pathways regulating cell adhesion and communication, both essential for embryo implantation. These findings advance our understanding of the molecular mechanisms underlying pregnancy success and failure in cattle.

## 1. Introduction

Pregnancy involves complex interactions between the fetus and the uterus [1]. Recent studies have shown that exosomes play an important regulatory role in physiological and pathological pregnancy by mediating information transmission between mother and fetus [2]. Exosomes are widely found in various cellular body fluids such as blood, urine, saliva, amniotic fluid, bronchoalveolar fluid and breast milk, and are rich in proteins, lipids, and miRNAs. These biologically active molecules, along with the secretion of exosomes, reach other cells and tissues through the circulatory system and participate in the remote regulation of cell communication [3]. Placental alkaline phosphatase exosomes in maternal plasma during pregnancy increased with the progression of pregnancy and had the highest concentration at term [4,5]. At present, many studies have shown that when the physiological state of the body changes, the exosome miRNAs in plasma will also change. By mapping the miRNAs expression profile of plasma exosomes in special physiological processes or pathological stages, researchers explore the function of miRNA to achieve the goal of assisting the treatment of diseases or optimizing the physiological state of the body through the regulation of miRNAs [6,7,8].

Exosomes are nanoscale (30–150 nm), membrane-bound vesicles secreted by diverse cell types under both physiological and pathological conditions. They are commonly characterized by their cup-shaped or spherical morphology under transmission electron microscopy (TEM) and their size distribution measured by nanoparticle tracking analysis (NTA) [9,10]. Moreover, exosomes are thought to mirror the molecular composition of their cells of origin.

They are widely considered as next-generation biomarkers for disease diagnosis, prognosis, and treatment [11]. Exosomes can be used as new predictive markers in the diagnosis of diseases, such as pregnancy disorders [12]. Menon et al. analyzed the circulating exosome miRNA profiles of human full-term pregnancy and premature birth, and they proposed that the circulating exosome miRNA in maternal blood may represent the bimolecular “fingerprint” of pregnancy progression [13]. Placenta-derived exosomes play a key role in the establishment of maternal immune tolerance, which is essential for a successful pregnancy [14,15]. In pigs, Chen et al. identified circulating miRNA profiles of serum exosomes from pigs in early gestation, miR-92b-3p and miR-17-5p may serve as potential circulating biomarkers for early pregnancy diagnosis [16]. In mice, exosomes in vitro embryo culture medium can improve the embryo attachment rate, increase the proliferation ability of mouse blastocyst cells, and reduce cell apoptosis [17]. In cattle, exosomes secreted by the uterus in the early luteal phase may play an important role in developmental competence of somatic cell nuclear transfer embryos [18]. Exosomes secreted from cattle conceptuses as well as endometria are involved in cell-to-cell interactions for conceptus implantation to the maternal endometrium [19].

On day seven of bovine pregnancy, the embryo is already capable of regulating gene expression in the endometrium and simultaneously inducing dynamic changes in the composition of uterine luminal metabolites [20,21]. Maternal recognition of pregnancy is initiated on days 16 to 17 followed by implantation and placentation [22]. This period is critical as approximately 40% of embryonic loss occurs between days 8 and 16 in cattle, coinciding with conceptus elongation and the initiation of maternal recognition of pregnancy [23]. Indeed, maternal recognition of pregnancy is initiated around day 16, a time when the conceptus secretes interferon-tau (IFNT) to signal its presence [24]. Notably, transcriptomic analysis has revealed no detectable differences in the endometrium on days 5, 7, or 13, but the expression of 764 genes is altered by day 16 [25]. Furthermore, previous plasma miRNA profiling studies have successfully used day 16 as a time point to identify putative biomarkers of pregnancy [26]. On day 16, after fertilization, when the uterus is in the receptive phase, miRNA profile in plasma exosomes may change and some miRNAs may affect the embryo implantation. In the current study, we investigated the miRNA profile in the plasma exosomes of pregnant and non-pregnant cows on day 16 after fertilization. The findings of this study provide a reference for screening and exploring the influence of exosomes miRNAs on embryo implantation and help to clarify the potential application of plasma exosomes in reproductive regulation.

## 2. Materials and Methods

### 2.1. Plasma Collection

Animal experiments were performed under the ethical guidelines and supervision of the Committee for Experimental Animals, Henan Academy of Agricultural Sciences. All heifers used in this study were approximately 13 months old and had never been inseminated before, making them primiparous, with no statistical difference in parity distribution. Blood was collected by evacuated blood tubes containing K2EDTA for each heifer on the 16th day after artificial insemination. Plasma was isolated from whole blood by centrifugation at 1000× *g* for 10 min and immediately flash-frozen in liquid nitrogen until subsequent analysis. Based on the result of ultrasound examination around day 40 after insemination, seven pregnant cows (YP) and seven non-pregnant cows (NP) were randomly selected for further study. Their plasma samples were used for exosome isolation.

### 2.2. Isolation of Exosomes from Plasma

The plasma samples were rapidly thawed in a water bath at 37 °C for approximately 5–10 min until completely thawed, then immediately transferred to a new centrifuge tube, centrifuged at 2000 *g* at 4 °C for 30 min. The supernatant has been carefully moved into a new centrifuge tube and centrifuged again at 10,000 *g* at 4 °C for 45 min to remove large vesicles. The supernatant was filtered by 0.45 μm filter (Millipore, Burlington, MA, USA), the resulting supernatant was centrifuged at 110,000 *g* for 70 min at 4 °C (Hitachi CP100MX, Hitachi, Tokyo, Japan). The pellet was suspended in 10 mL pre-cooling PBS and centrifuged at 110,000 *g* for 70 min at 4 °C again. Finally, the supernatant was removed and re-suspended in 200 μL pre-cooled 1 × PBS and stored at −80 °C.

### 2.3. Transmission Electron Microscopy

A 10-μL aliquot was applied onto a 200-mesh carbon-coated copper grid (Ted Pella, Inc., Redding, CA, USA) for 1 min, negatively stained with 2% phosphotungstic acid for 1 min, and excess liquid was blotted with filter paper. The grids were then air-dried at room temperature for 15 min. Samples were visualized under a transmission electron microscope (Hitachi HT-7700, Tokyo, Japan).

### 2.4. Nanoparticle Tracking Analysis

An aliquot (10 μL) of exosome samples was diluted to a total volume of 30 μL with PBS (3-fold dilution). The diluted samples were analyzed using a ZetaView Nanoparticle Tracking Analyzer (Particle Metrix, Inning am Ammersee, Germany) with a 488 nm laser, 1.0 KPa sample pressure, and 0.3 ms minimum width to determine the average particle diameter and concentration.

### 2.5. Small RNA Library Construction

Total RNA was isolated from plasma-derived exosomes with the miRNeasy Serum/Plasma Kit (Qiagen, Hilden, Germany) following the manufacturer’s protocol. The experimental procedures, including library preparation and sequencing, were conducted following the standard protocols provided by Illumina. Small RNA sequencing libraries were constructed using TruSeq Small RNA Sample Prep Kits (Illumina, San Diego, CA, USA) and subsequently sequenced on an Illumina Hiseq2500 (Illumina, San Diego, CA, USA), and the sequencing read length was Single-end 50 bp. Small RNA sequencing and analysis were performed by LC Sciences (LC Sciences, Hangzhou, China).

### 2.6. Quality Control and miRNA Alignment

Raw reads were subjected to an in-house program, ACGT101-miR (v4.2) (LC Sciences, Hangzhou, China), to remove adapter dimers, junk, low complexity, common RNA families (rRNA, tRNA, snRNA, snoRNA) and repeats. The analysis process is as follows: clean reads were obtained by removing reads with low quality, 5′ primer contaminants and poly (A). Reads without 3′ adapter, insert tag, and sequences with base lengths of 18–26 nt were retained. The length distribution of the clean reads in the reference genome (ARS-UCD 1.2) was determined. Clean reads were mapped in miRBase v21 [27] using Bowtie v1.1.1. The unmapped reads were mapped to multiple databases, including mRNA, Rfam 11.0, and genomic DNA using Bowtie v1.1.1 [28] (Table S1).

### 2.7. MiRNA Expression Analysis

Clean data used ACGT101-miR (v4.2) to identify small RNAs and calculate the expression level of miRNA identified in each sample [29]. The expression levels of miRNA were normalized by ACGT101-miR (v4.2) [30]. Differential expression analysis between two groups was conducted. The two-tailed Student’s *t*-test was used to assess the statistical significance between the two groups and obtain the raw *p*-values in R (v3.6.0). PBH values were then calculated using Benjamini–Hochberg correction for multiple testing in R. However, none of the miRNAs met the adjusted significance threshold (PBH < 0.05), likely due to the modest sample size (*n* = 7 per group). Therefore, adjusted *p*-values were not used as filtering criteria. The miRNAs with *p*-value < 0.05 and |log2FoldChange| > 1 were considered as significantly differentially expressed miRNAs. TargetScan (v5.0) [31] (Table S4) and miRanda (v3.3a) [32] (Table S5) were used to predict the target genes of miRNAs with significant differences. Target genes with context score percentile less than 50 were removed from TargetScan algorithm, and target genes with maximum free energy greater than −10 were removed from miRanda algorithm. Target gene prediction for the differentially expressed miRNAs was conducted using TargetScan_score ≥ 50 and miranda_Energy ≤ −10. Gene Ontology (GO) enrichment and Kyoto Encyclopedia of Genes and Genomes (KEGG) pathway enrichment analyses were performed on the predicted target genes using the OmicStudio Classic package (LC Sciences, Hangzhou, China) within R (v4.1.3), based on the hypergeometric distribution. Given the substantial number of enriched entries and pathways identified through GO (Table S2) and KEGG (Table S3) analyses, we subsequently selected the top 50 GO entries and the top 20 KEGG pathways for further analysis and visualization.

## 3. Results

### 3.1. Exosome Isolation and Characterization

**Figure 1:**
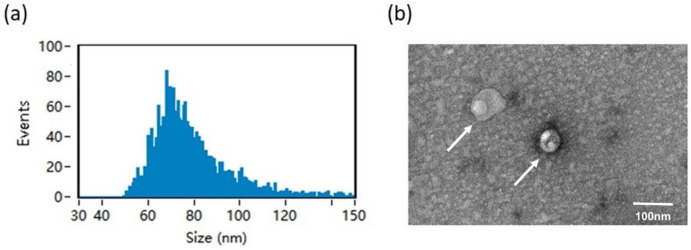
Isolation and characterization of plasma exosomes. (**a**) NTA showing the particle size distribution of a representative plasma exosome sample. The peak particle size was 78.62 nm, falling within the typical exosomal range (30–150 nm). (**b**) TEM image of a representative plasma exosome sample, revealing typical cup-shaped morphology. Arrows indicate exosomes isolated from plasma. Scale bar = 100 nm.

One of the plasma exosomes was randomly selected for the identification using NTA and TEM. The results showed that the isolated vesicles had typical exosome sizes, with the most widely distributed particle size of 78.62 nm (Figure 1a). The result of TEM showed that the vesicles were bowl-shaped, which has the classic exosome morphology (Figure 1b).

### 3.2. Differential miRNA Expression Analysis

**Figure 2:**
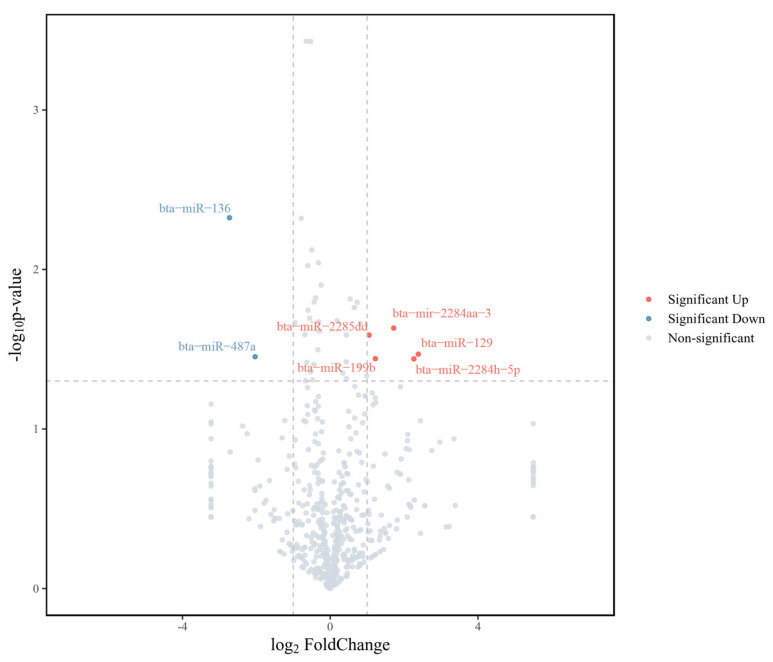
Volcano plot of differentially expressed miRNAs between the two groups. The two vertical dashed lines indicate a threshold of |Log_2_FC| > 1, and the horizontal dashed line indicates a threshold of *p*-value < 0.05. Red dots and blue dots denote miRNAs were upregulated and downregulated in the group of pregnant cows, respectively. The significance cut-off was set to a *p*-value of 0.05.

After obtaining the raw data, we performed filtering and trimming were performed to retain high-quality sequences for downstream analysis. A total of 2267 unique miRNAs were differentially expressed between the two groups. Among these, seven miRNAs were significantly differentially expressed between the pregnant and non-pregnant groups (*p*-value < 0.05 and |log2FoldChange| > 1) (Figure 2). Benjamini–Hochberg correction was applied to calculate adjusted *p*-values (PBH). Due to the modest sample size, none of the miRNAs met the adjusted significance threshold (PBH < 0.05). Nevertheless, the consistent direction of change across the seven miRNAs, together with the biological relevance of the enriched pathways, suggests that these miRNAs may still be functionally relevant. Five miRNAs were upregulated and two miRNAs downregulated in the group of pregnant cows. Specifically, five miRNAs (bta-mir-2284aa-3, bta-miR-2285dd, bta-miR-129, bta-miR-199b, and bta-miR-2284h-5p) were upregulated, and two miRNAs (bta-miR-136 and bta-miR-487a) were downregulated in the pregnant cows (Table 1).

### 3.3. GO Enrichment and KEGG Pathway Analyses of Predicted Target Genes of the Differentially Expressed miRNAs

**Figure 3:**
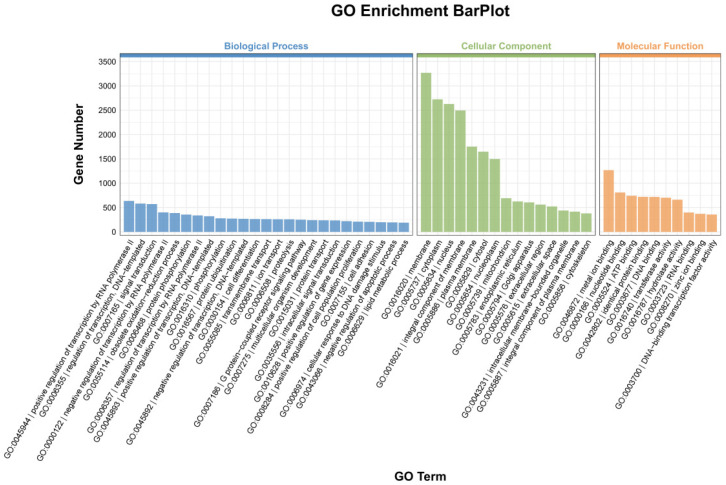
GO analysis of predicted target genes of differentially expressed miRNAs.

Using the seven differentially expressed miRNAs, 5828 non-repeating target genes were predicted. GOseq was employed to annotate the GO function of the predicted target genes and assign them to GO biological process (BP), cell component (CC) and molecular function (MF) categories (Figure 3). Due to the uneven distribution of enriched terms across categories (BP > CC > MF), the top 25 BP terms, top 15 CC terms, and top 10 MF terms are shown, ranked by the number of annotated target genes. The top five GO terms for each category are as follows: BP—“positive regulation of transcription by RNA polymerase II”, “regulation of transcription, DNA-templated”, “signal transduction”, “negative regulation of transcription by RNA polymerase II” and “obsolete oxidation-reduction process”. CC—“membrane”, “cytoplasm”, “nucleus”, “integral component of membrane”, “plasma membrane”. MF—“metal ion binding”, “nucleotide binding”, “ATP binding”, “identical protein binding”, “DNA binding” Some functional pathway and signal pathway such as “apoptotic process”, “in utero embryonic development” and “cell junction” were related to the embryo implantation. For KEGG analysis, 176 terms were significant. Some KEGG pathways in the top 20 terms, such as “Rap1 signaling pathway”, “MAPK signaling pathway”, “Adherens junction”, and “Cell adhesion molecules” were related to embryo implantation and endometrial development [33,34,35,36] (Table 2). Therefore, we suggest that these rich signaling pathways reflect the biological processes of pregnancy and their potential regulatory mechanisms.

## 4. Discussion

Exosomes have significant effects on embryo development, implantation and endometrial receptivity. The identification of exosomes relies on a comprehensive analysis combining biological physics and molecular characteristics. Our TEM and NTA results show that exosomes are cup-shaped, with an average diameter of 78.62 nm, which is highly consistent with previous reports on exosomes derived from bovine plasma. In the implantation window, embryos and endometrium can communicate with each other in the form of secreting exosomes in a complex way, and the miRNAs in exosomes may affect the embryo implantation rate [37]. Between cows with normal embryos and those with dead embryos there are significant differences in the miRNA expression profiles in plasma exosomes [38]. Moreover, a recent study demonstrated that circulating miR-126-3p may serve as a biomarker for early pregnancy diagnosis in cattle, and that EV-associated miRNAs can be reliably detected in plasma [39]. These findings support the relevance of exosomal miRNAs as indicators of pregnancy status.

In the current study, miRNAs expression profiles in plasma exosomes of pregnant and non-pregnant cows on day 16 were characterized. Seven differentially expressed miRNAs were identified. Their predicted target genes were significantly enriched in several GO categories and KEGG pathways associated with biological processes or pathways associated with embryo implantation and endometrial development. These results of GO analysis such as cell adhesion and cell differentiation were related to embryo implantation and endometrial development [40,41]. In addition, among the KEGG analysis results, several signaling pathways, such as the MAPK signaling pathway, Ras signaling pathway, PI3K-Akt signaling pathway, and Rap1 signaling pathway provided more evidence that the differentially expressed miRNAs identified in this study were involved in the embryo implantation and endometrial development [42,43,44,45]. The MAPK signaling pathway has been shown to be related to focal adhesion in many experiments [46,47]. Ras-MAPK pathway activation governs trophectoderm specification, which is a process operational in embryonic stem cell models and preimplantation mouse embryos [48]. The PI3K-Akt signaling pathway is associated with cell proliferation, apoptosis, and autophagy [49,50], and it was demonstrated in mice that the activation of this pathway is involved in glucose metabolism and embryo survival [51]. The Rap1 signaling pathway has many important biological functions such as control of cell adhesion, cell junction, and cell proliferation [52,53,54]. In addition, studies have analyzed the differential expression of miRNAs in uterine fluid secreted by patients with repeated implantation failure and fertile women. These studies found that the adherens junction and cell adhesion pathways were also enriched by differentially expressed surface adhesion miRNAs [55,56].

Among the miRNAs with significant differences, we identified that bta-miR-136, bta-miR-199b and bta-miR-382-3p are associated with embryo implantation and development.

MiR-136 showed the most significant difference, and miR-136 was significantly downregulated. Studies have shown that miR-136 promotes vascular myocyte proliferation by targeting PPP2R2A through the ERK1/2 pathway [57]. Endometrial epithelial cell apoptosis is a sign of endometrial receptivity [58,59]. We conjecture that miR-136, as the most distinct miRNA that can regulate cell proliferation and apoptosis, has a great possibility to play a role in the process of embryo implantation.

The regulation of miR-199b is involved in many biological processes in animals, and it has been reported that miR-199b may be used as a biomarker for locally advanced rectal cancer (LARC) [60], Kusama also screened significantly upregulated miR-199b in the uterine fluid of cows at the 7th day of gestation, which was the same as the conclusion of this experiment [61]; therefore, miR-199b should also attract our attention. Among the miRNAs with significant differences, we found some miRNAs involved in embryo implantation, which were consistent with previous literature reports. Due to the small number of differential miRNAs screened, we recovered some differential miRNAs after relaxing the threshold of FC. Both miR-199a-3p and miR-199b can control apoptosis and inflammation by targeting IKKβ to regulate the NF-κB pathway [62,63]. NF-κB is an inflammatory regulator, and Zhao’s article suggests that placental exosome mediated bta-miR-499 may mediate inflammation in cows early in pregnancy by targeting the NF-κB signaling pathway [64].

At present, there are not many studies on the role of m in the field of reproduction. MiR-382-3p can guide apoptosis by targeting the RhoC/ROCK1 signaling pathway [65]. Recently demonstrated that the lncRNA PSMB8-AS1 functions as a ceRNA by sponging miR-382-3p, thereby regulating cell proliferation, migration, and apoptosis via the miR-382-3p/BCAT1 axis in glioma cells [66], supporting a broader role for miR-382-3p in cell behavioral regulation. We speculate miR-382-3p can be used as a candidate miRNA for further validation of its function.

We acknowledge several limitations in this study. First, exosome characterization did not fully comply with MISEV2018 guidelines, as Western blotting for exosomal markers (CD9, CD63, TSG101) was not performed due to sample depletion. Nevertheless, NTA and TEM confirmed that the isolated vesicles were within the exosomal size range (30–150 nm) and displayed typical cup-shaped morphology, providing strong evidence of successful exosome enrichment. Second, our sample size was modest (*n* = 7 per group). After Benjamini–Hochberg correction for multiple testing, no miRNA reached PBH < 0.05. Therefore, we used raw *p*-values combined with a fold-change threshold (|log2FC| > 1) to identify candidate miRNAs—an exploratory approach that increases the risk of false positives. Third, due to sample depletion and lack of an independent cohort, qPCR validation and confirmation of EV-specific signals could not be performed and are planned for future studies. Despite these limitations, the seven miRNAs identified here warrant further investigation.

Despite these limitations, our study provides a valuable preliminary profile of plasma exosomal miRNAs during early pregnancy in cattle. The candidate miRNAs identified—particularly bta-miR-136, bta-miR-199b, and bta-miR-382-3p—warrant further investigation in larger cohorts and functional studies to elucidate their roles in maternal–fetal communication and embryo implantation.

## 5. Conclusions

In conclusion, this study identified seven differentially expressed miRNAs in plasma exosomes between pregnant and non-pregnant cows on day 16. Among these, bta-miR-136, bta-miR-199b, and bta-miR-382-3p emerged as candidate miRNAs for further investigation. Our study provides an experimental basis for screening and exploring plasma exosome miRNAs involved in cattle pregnancy. Future studies should validate these candidate miRNAs in larger independent cohorts and elucidate their functional roles in endometrial or trophoblast cell lines using miRNA mimic or inhibitor transfection assays. The potential of these miRNAs as biomarkers for early embryonic death—a major cause of pregnancy loss in cattle that often occurs before day 30 of gestation—also warrants further investigation. Longitudinal profiling of plasma exosomes miRNAs from day 16 through early gestation may help identify miRNA signatures associated with subsequent pregnancy failure. Such efforts could ultimately contribute to improved reproductive management and early pregnancy diagnosis in dairy and beef herds.

## Figures and Tables

**Table 1:** Significantly different miRNAs expression levels in pregnant cow groups.

miRNA	Regulation	log2 FoldChange	*p*-Value
bta-miR-136	down	−2.73	0.0047
bta-miR-2284aa-3	up	1.72	0.0233
bta-miR-2285dd	up	1.05	0.0258
bta-miR-129	up	2.39	0.0340
bta-miR-487a	down	−2.03	0.0352
bta-miR-199b	up	1.22	0.0362
bta-miR-2284h-5p	up	2.27	0.0363

**Table 2:** Enriched pathways (top 20) of differentially expressed miRNA-predicted target genes in exosomes.

Term	ID	*p*-Value	q-Value
MAPK signaling pathway	bta04010	2.977 × 10^−17^	9.466 × 10^−15^
Pathways in cancer	bta05200	3.700 × 10^−11^	5.883 × 10^−9^
Proteoglycans in cancer	bta05205	8.784 × 10^−11^	9.311 × 10^−9^
MicroRNAs in cancer	bta05206	1.473 × 10^−9^	1.171 × 10^−7^
Ras signaling pathway	bta04014	3.104 × 10^−9^	1.974 × 10^−7^
Rap1 signaling pathway	bta04015	4.933 × 10^−8^	2.615 × 10^−6^
Phosphatidylinositol signaling system	bta04070	4.065 × 10^−7^	1.846 × 10^−5^
Fluid shear stress and atherosclerosis	bta05418	1.212 × 10^−6^	4.315 × 10^−5^
HIF-1 signaling pathway	bta04066	1.221 × 10^−6^	4.315 × 10^−5^
Human cytomegalovirus infection	bta05163	1.482 × 10^−6^	4.643 × 10^−5^
Endocytosis	bta04144	1.606 × 10^−6^	4.643 × 10^−5^
Cellular senescence	bta04218	1.775 × 10^−6^	4.704 × 10^−5^
Human T-cell leukemia virus 1 infection	bta05166	1.988 × 10^−6^	4.863 × 10^−5^
Cell cycle	bta04110	2.621 × 10^−6^	5.854 × 10^−5^
Rheumatoid arthritis	bta05323	2.762 × 10^−6^	5.854 × 10^−5^
Choline metabolism in cancer	bta05231	3.482 × 10^−6^	6.919 × 10^−5^
Oxytocin signaling pathway	bta04921	5.096 × 10^−6^	9.532 × 10^−5^
AGE-RAGE signaling pathway in diabetic complications	bta04933	7.875 × 10^−6^	0.0001272
Melanogenesis	bta04916	7.875 × 10^−6^	0.0001272
Human immunodeficiency virus 1 infection	bta05170	8.171 × 10^−6^	0.0001272

## Data Availability

The original contributions presented in this study are included in the article/Supplementary Material. Further inquiries can be directed to the corresponding author.

## Supplementary Materials

The following supporting information can be downloaded at: https://www.mdpi.com/article/10.3390/biology15110863/s1, Table S1. Per-sample sequencing quality control metrics. Table S2. Overview of reads from raw data to cleaned sequences. Table S3. TargetScan predicted target genes result. Table S4. Miranda predicted target genes result. Table S5. The miRNAs differ significantly between the two groups of cattle (P-0.05).
